# Integrating single-cell RNA-seq to identify fibroblast-based molecular subtypes for predicting prognosis and therapeutic response in bladder cancer

**DOI:** 10.18632/aging.206021

**Published:** 2024-07-18

**Authors:** Jia Wang, Zhiyong Tan, Yinglong Huang, Charles Li, Peiqin Zhan, Haifeng Wang, Haihao Li

**Affiliations:** 1 The Second Clinical Medical College, Kunming Medical University, Kunming, China; 2 Department of Endocrinology, The Second Affiliated Hospital of Kunming Medical University, Kunming, China; 3 Department of Urology, The Second Affiliated Hospital of Kunming Medical University, Kunming, China; 4 Core Facility for Protein Research, Chinese Academy of Sciences, Beijing, China; 5 Zhongke Jianlan Medical Research Institute, Beijing, China; 6 Zhejiang Institute of Integrated Traditional and Western Medicine, Hangzhou, China

**Keywords:** scRNA-seq, bladder cancer, prognostic signature, fibroblast cell, immunotherapy response

## Abstract

Background: Bladder cancer (BLCA) is a highly aggressive and heterogeneous disease, posing challenges for diagnosis and treatment. Cancer immunotherapy has recently emerged as a promising option for patients with advanced and drug-resistant cancers. Fibroblasts, a significant component of the tumor microenvironment, play a crucial role in tumor progression, but their precise function in BLCA remains uncertain.

Methods: Single-cell RNA sequencing (scRNA-seq) data for BLCA were obtained from the Gene Expression Omnibus database. The R package “Seurat” was used for processing scRNA-seq data, with uniform manifold approximation and projection (UMAP) for downscaling and cluster identification. The FindAllMarkers function identified marker genes for each cluster. Differentially expressed genes influencing overall survival (OS) of BLCA patients were identified using the limma package. Differences in clinicopathological characteristics, immune microenvironment, immune checkpoints, and chemotherapeutic drug sensitivity between high- and low-risk groups were investigated. RT-qPCR and immunohistochemistry validated the expression of prognostic genes.

Results: Fibroblast marker genes identified three molecular subtypes in the testing set. A prognostic signature comprising ten genes stratified BLCA patients into high- and low-score groups. This signature was validated in one internal and two external validation sets. High-score patients exhibited increased immune cell infiltration, elevated chemokine expression, and enhanced immune checkpoint expression but had poorer OS and a reduced response to immunotherapy. Six sensitive anti-tumor drugs were identified for the high-score group. RT-qPCR and immunohistochemistry showed that CERCAM, TM4SF1, FN1, ANXA1, and LOX were highly expressed, while EMP1, HEYL, FBN1, and SLC2A3 were downregulated in BLCA.

Conclusion: A novel fibroblast marker gene-based signature was established, providing robust predictions of survival and immunotherapeutic response in BLCA patients.

## INTRODUCTION

Bladder cancer (BLCA) is the 10th most common cancer worldwide, with uroepithelial carcinoma accounting for about 90% of cases. In 2020, there were approximately 573,000 new cases and 213,000 deaths globally [1, 2]. Despite various treatments, the prognosis for BLCA patients remains poor, with a median overall survival (OS) of just 14 months [3]. Chemotherapy is a key first-line treatment, but many patients eventually develop chemoresistance and experience tumor recurrence. Potential mechanisms behind this include enhanced chemoresistance in bladder cancer stem cells (CSCs) and the regulatory inactivation of oncogenes and tumor suppressor genes [4, 5].

Immune checkpoint inhibitors (ICIs) have become important for treating advanced and cisplatin-resistant BLCA, with PD-1/PD-L1 inhibitors approved for first- and second-line therapy. However, only a minority of patients benefit from these treatments [6, 7]. Biomarkers like tumor mutation burden (TMB) and immune checkpoint expression are used to predict immunotherapy response [8, 9], but do not fully capture the tumor microenvironment (TME) heterogeneity. Therefore, a tool to accurately predict BLCA prognosis and immunotherapy response is desperately needed.

The TME includes various immune cells, stromal cells, extracellular matrix (ECM) molecules, and cytokines [10]. Changes in the TME composition can significantly affect tumor progression, invasion, and immunotherapy response [11, 12]. Fibroblasts, the main stromal cells in the TME, are transformed into cancer-associated fibroblasts (CAFs) by tumor cell-generated paracrine growth factors [13]. CAFs play roles in tumor cell proliferation, angiogenesis, ECM remodeling, and anti-tumor immunity [14]. They can secrete immunosuppressive cytokines like IL-6, CXCL1, and CXCL12, promoting immune escape or forming physical barriers to immune cell entry through ECM remodeling [15, 16]. BLCA patients with an immune-excluded phenotype due to CAFs and ECM show decreased responses to nivolumab, while muscle-invasive BLCA patients with low CAF levels are more likely to achieve complete pathological remission with neoadjuvant chemotherapy [17].

Given the critical role of fibroblasts in anti-tumor immunity, further research on fibroblasts is essential for new BLCA treatments. Single-cell RNA sequencing (scRNA-seq) allows for the identification of individual immune cell subpopulations in the TME, providing insights into immune cell heterogeneity and molecular characteristics [18]. This study aims to identify fibroblast marker genes and molecular subtypes and establish a prognostic signature by integrating scRNA-seq and bulk RNA-seq data. We evaluated the correlation between the signature and prognosis, clinicopathological features, and immunotherapy response in BLCA patients. Our results offer promising biomarkers and potential therapeutic targets for BLCA patients.

## MATERIALS AND METHODS

### Data source and processing

A total of 957 BLCA samples were included in this study. The scRNA-seq dataset GSE135337 containing seven primary tumor samples was obtained from the Gene Expression Omnibus (GEO) database (http://www.ncbi.nlm.nih.gov/geo/), and it was utilized to identify marker genes of fibroblasts. The TCGA-BLCA dataset (411 samples) was merged with the GSE13507 dataset (165 samples), which were downloaded from UCSC Xena (https://xenabrowser.net/) and the GEO database, respectively. Batch effects were corrected with the “limma” and “sva” packages [19], which were used to construct the prognostic signature. To evaluate the predictive value of the prognostic signature for the effectiveness of immunotherapy in patients, the transcriptomic and matched clinical data of patients who underwent anti-PD1 and anti-PD-L1 treatment from the GSE78220 dataset (n=26) and IMvigor210 cohort (n=348) were collected from GEO database and “IMvigor210CoreBiologies” package (http://research-pub.gene.com/IMvigor210CoreBiologies) [20]. This study was approved by the Ethics Committee of the Second Affiliated Hospital of Kunming Medical University (KYD213238).

### scRNA-seq analysis

The scRNA-seq data were screened and analyzed using the R package “Seurat” [21]. Firstly, we performed quality control of the scRNA-seq data with the following criteria: 1) exclusion of genes that appeared in fewer than five cells; 2) removal of cells that expressed fewer than 300 genes; 3) elimination of cells with mitochondrial gene expression above 10%; 4) retention of cells with ribosomal genes > 3% and 5) inclusion of cells with hemoglobin genes < 0.1%. We then used the R package “harmony” to reduce batch effects between samples. Subsequently, we normalized the scRNA-seq data using the “ScaleData” and performed principal component analysis (PCA), and the uniform manifold approximation and projection (UMAP) [22] function to reduce the dimensionality. We employed the “FindAllMarkers” function to identify differentially expressed genes in various clusters. Finally, cells were clustered by a resolution of 0.8 and cell annotation was performed by the R package “singleR” [23] combined with manual adjustment.

### Consensus clustering analysis

Based on ten fibroblast marker genes, consensus clustering analysis was performed on the samples of the testing set with the R package “ConsensusClusterPlus” [24] to identify fibroblast subtypes. To ensure the stability of the clustering process, we employed a resampling rate of 80% and 1000 repeats. Subsequently, we selected cluster-effective results as the subtypes of BLCA patients based on survival analysis and differential expression of marker genes.

### Gene sets variation analysis (GSVA) and immune cell infiltration analysis

For evaluation of differences in pathways between the three subtypes, we downloaded the HALLMARK, Kyoto Encyclopedia of Genes and Genomes (KEGG), and Reactome pathways from the MSigDB database (http://www.gsea-msigdb.org/gsea/index.jsp) and scored them utilizing the R package “GSVA” [25]. Heatmap plots were generated via the “pheatmap” package. To compare the immune cell infiltration between fibroblast subtypes, the immune score, stromal score, and ESTIMATE score of BLCA patients were measured using the R package “ESTIMATE” [26], and the fraction of immune cell infiltration was calculated by “ssGSEA” function of the R package “GSVA”.

### Construction of prognostic signature based on fibroblast cell marker genes

Firstly, difference analysis was performed for each of the three subtypes, and genes that exhibited a |log2 (fold change)|>1 and p-value<0.05 were considered as differentially expressed genes (DEGs). These DEGs were pooled to create a gene set, which we then subjected to the Gene Ontology (GO)/KEGG analysis using the R package “clusterprofiler” [27]. Next, we conducted univariate Cox regression analysis on all DEGs to identify the ten signature genes with the smallest p-value associated with prognosis. Subsequently, we performed Consensus clustering analysis on the signature genes to obtain two molecular subtypes. Finally, the final signature scores were calculated by the GSVA method based on ten signature genes, and prognostic signatures were established. In addition, the Kaplan-Meier survival curve was generated using the R package “survminer” (https://CRAN.R-project.org/package=survminer) to compare the differences in OS between high- and low-score groups.

### Relationship between prognostic signature and immunotherapy

To elucidate the relationship between risk score and tumor immune microenvironment, correlation analysis was used to assess the relationship between risk score and immune cell infiltration, as well as 50 hallmark pathways. A significant correlation was defined as p<0.05. Moreover, the expression of multiple chemokines and immune checkpoints was compared between high- and low-score groups to reveal the relationship between risk score and immunotherapy. As TMB is closely related to immunotherapy efficacy, we analyzed the TMB levels between the two groups by the R package “maftools” [28]. In addition, the predictive ability of risk scores for immunotherapy was verified in the internal validation set GSE13507 and the external validation set GSE78220 and IMvigor210, respectively.

### Drug sensitivity analysis

To investigate potential treatment options for immunotherapy-insensitive patients, we directed our attention toward targeted therapies. We estimated the 50% maximum inhibitory concentration (IC_50_) of each sample to multiple anti-cancer drugs using the R package “pRRophetic” [29]. We then compared the differences in IC_50_ values between the high- and low-score groups, where a higher IC_50_ value implies lower sensitivity to treatment.

### Quantitative real-time PCR (RT-qPCR) assays

To validate mRNA levels, we performed RT-qPCR according to the manufacturer’s instructions. We purchased a normal bladder uroepithelial cell line (SV-HUC-1) and BLCA cell lines (UM-UC-3, RT4, T24, 5627, SW780, and J82) were purchased from the Shanghai Cell Bank of the Chinese Academy of Sciences and cultured them in Roswell Park Memorial Institute (RPMI) 1640 medium supplemented with 10% fetal bovine serum. We extracted total RNA from BLCA and normal bladder uroepithelial cells using TRIzol reagent (Life Technology, CA, USA) and reverse transcribed it into cDNA using PrimeScript RT Master Mix (Takara, Tokyo, Japan), following the manufacturer’s instructions. Glyceraldehyde 3-phosphate dehydrogenase (GAPDH) was used as an internal control. The 2^-ΔΔCt^ method was used to determine relative gene expression levels. Supplementary Table 1 lists all mRNA primers used.

### Immunohistochemistry (IHC) staining

A total of 24 pairs of paraffin-embedded BLCA and adjacent samples were obtained from the Second Affiliated Hospital of Kunming Medical University, with approval from the ethics committee and written informed consent from each patient for the use of their materials. The tissue slices were baked at 65° C for 2 hours, followed by dewaxing and antigen retrieval. Endogenous enzymes were blocked using a 3% hydrogen peroxide solution for 10 minutes at room temperature. The slices were rinsed three times with phosphate buffer solution (PBS) for 3 minutes each time and then blocked with bovine serum albumin. The primary antibody was applied and incubated overnight at 4° C. After washing the slices three times with PBS for 5 minutes each time, the secondary antibody was applied and incubated at 37° C for 30 minutes. Diaminobenzidine was used for color rendering for 5-10 minutes, followed by hematoxylin redyes for 3 minutes. The slices were observed under a microscope, and the integrated optical density (IOD) was measured using Image Pro Plus 6.0 image software. The relative expressions were presented as the average optical density (IOD/positive staining area).

### Statistical analysis

All statistical analyses and data visualization were performed using the R software (Version 4.1.1) and GraphPad Prism (Version 9.0). We used Pearson correlation analysis to assess correlations between two continuous variables. The Wilcoxon test was employed to compare differences between two variables, and the Kruskal-Wallis test was used to compare multiple variables. Statistical significance was set at a two-sided p-value < 0.05.

### Availability of data and materials

The analyzed datasets generated during the study are available from the corresponding author upon reasonable request.

## RESULTS

### Identification of fibroblast marker gene expression profiles

**Figure 1:**
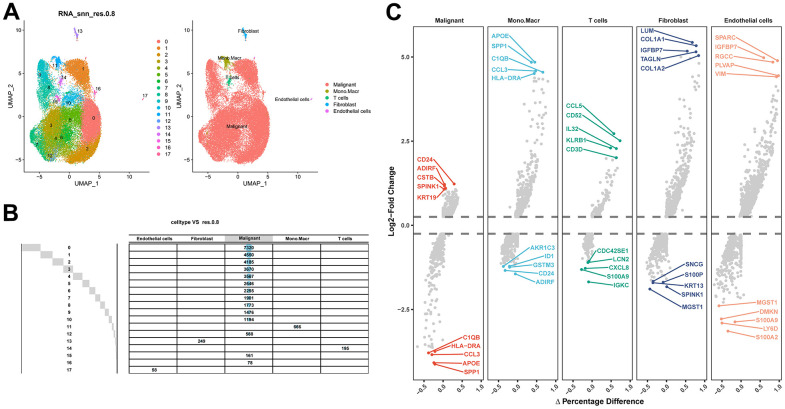
**Identification of fibroblast marker genes based on scRNA-seq data.** (**A**) 18 cell populations and 5 cell types visualized by the UMAP algorithm. (**B**) Amount of cells and attribution of each cell type. (**C**) Identification of top 5 differential genes for each cell type by FindAllMarkers.

**Figure 2:**
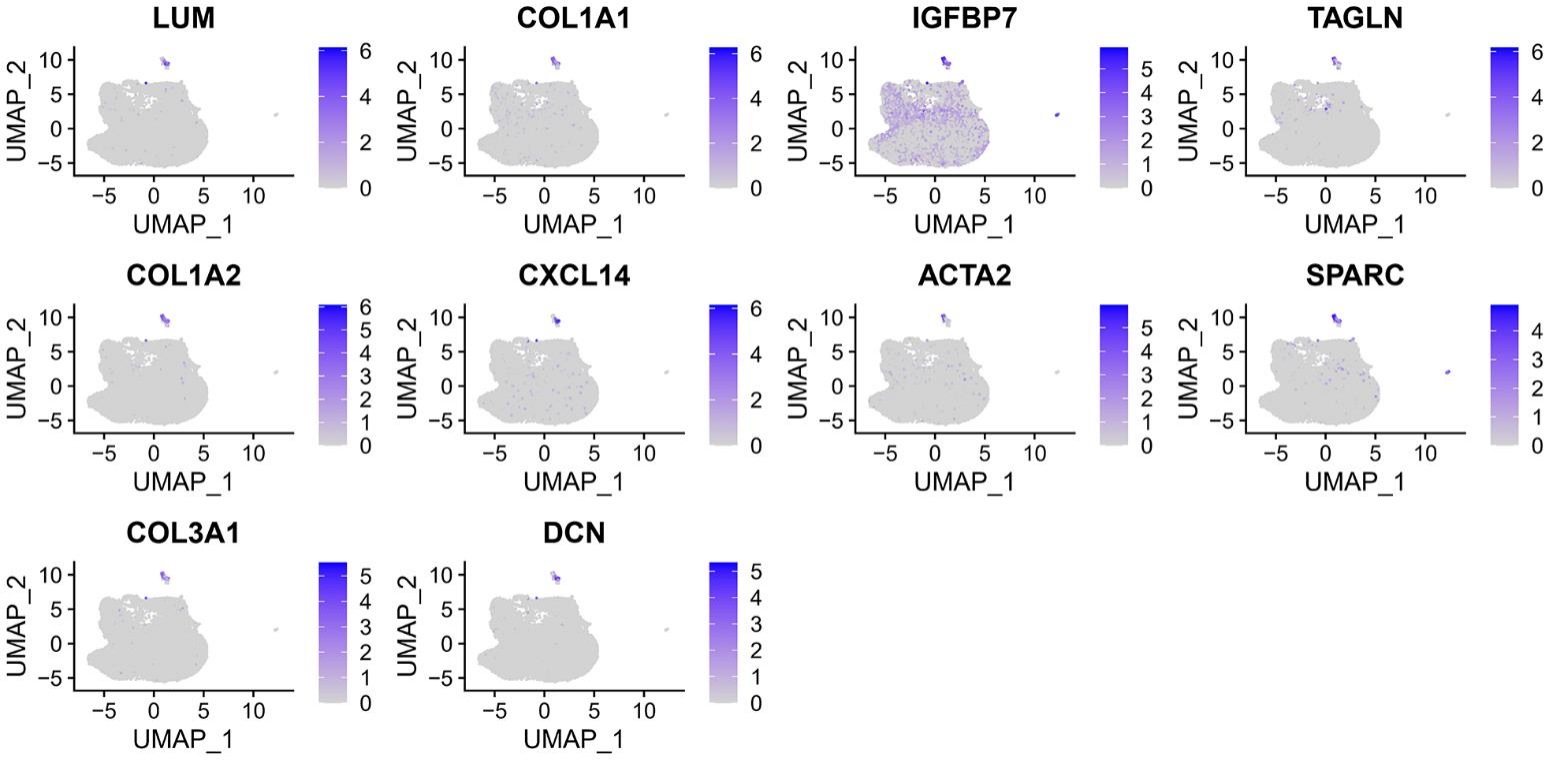
The expression of 10 fibroblast marker genes.

**Figure 3:**
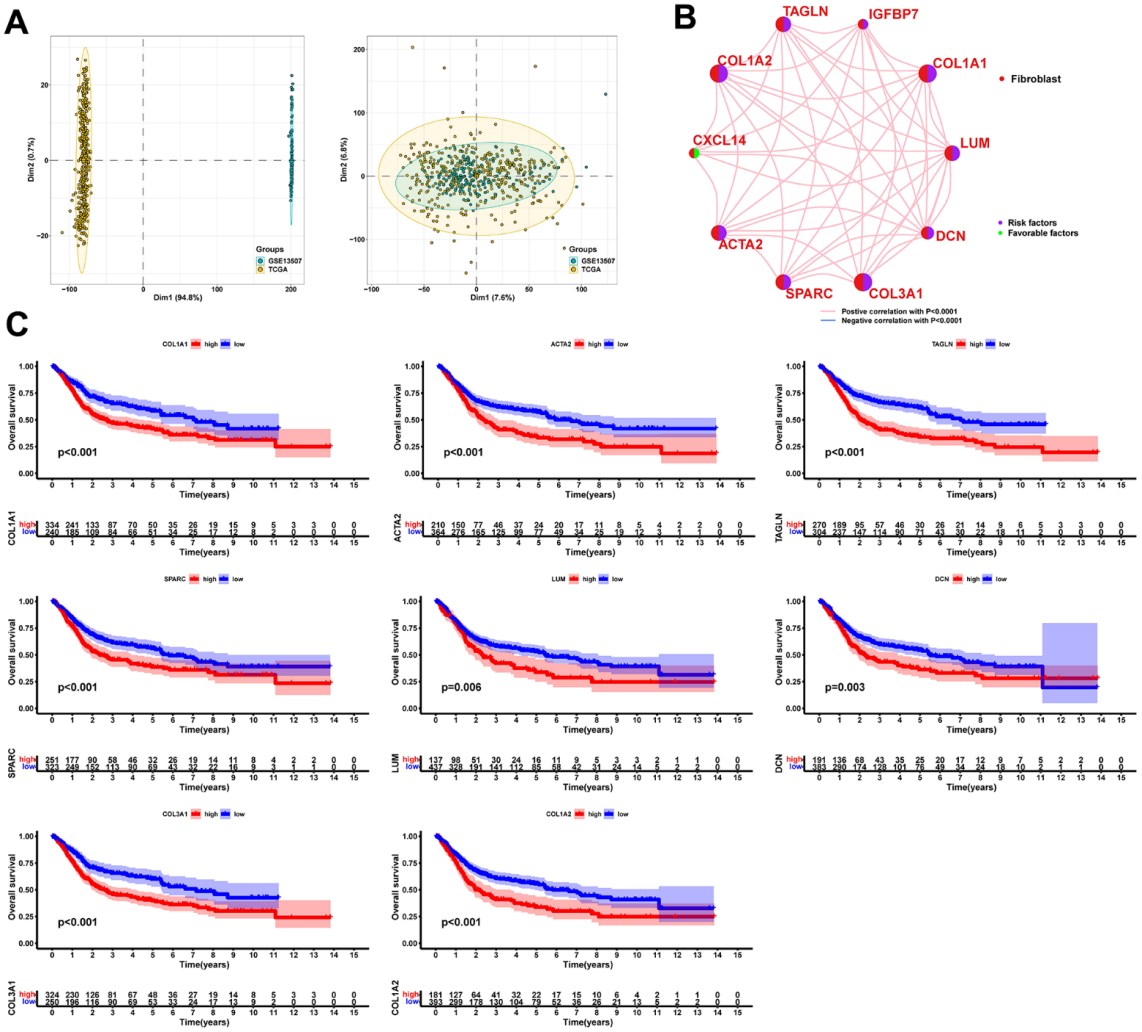
**Significant correlation between fibroblast marker genes and prognosis of BLCA patients.** (**A**) PCA plots of the testing set data before and after integration. (**B**) Correlation analysis and univariate regression analysis between fibroblast marker genes. Lines indicate a correlation between genes and p<0.0001; purple indicates a prognostic risk factor and green indicates a protective factor. (**C**) Kaplan-Meier curves for each marker gene with p<0.05.

After filtering the scRNA-seq data, we obtained information on the range of detected gene numbers, the number of transcripts sequenced, the percentage of mitochondrial/ribosomal/hemoglobin content in each sample, and the top 25 genes with the highest rate (Supplementary Figure 1). After reducing the dimensions, we identified 18 clusters and annotated them into five core cell types: T cells, malignant cells, monocytes, macrophages, fibroblasts, and endothelial cells. 21160 genes and 36532 cells were used for further analysis (Figure 1A). Notably, cluster 13 was defined as a fibroblast subpopulation. Figure 1B depicts the number of cells in each cell type, where the fibroblast subpopulation consists of 249 cells. Differential gene expression analysis was conducted for each cell type, and Figure 1C illustrates the top 5 up and down-regulated genes in each cell type. A detailed list of differential genes is provided in Supplementary Table 2. Among the fibroblast marker genes, we selected the top 10 differentially expressed genes, namely LUM, COL1A1, IGFBP7, TAGLN, COL1A2, CXCL14, ACTA2, SPARC, COL3A1, and DCN, and visualized their expression patterns in cells using “FeaturePlot” (Figure 2). Additionally, we observed a positive correlation between these marker genes (p<0.0001), and the results of univariate regression analysis revealed that all nine marker genes, except for CXCL14, were prognostic risk factors (Figure 3B). Following batch effect removal, the TCGA-BLCA and GSE13507 datasets were combined as the testing set, which comprised 17147 genes and 576 samples (Figure 3A). The testing set was divided into gene high- and low-expression groups according to the best cutoff value, and our analysis demonstrated that the OS of BLCA patients was significantly worse (p<0.05) in the high-expression group of the majority of marker genes (Figure 3C).

### Correlation between fibroblast marker gene subtypes and tumor immune microenvironment

**Figure 4:**
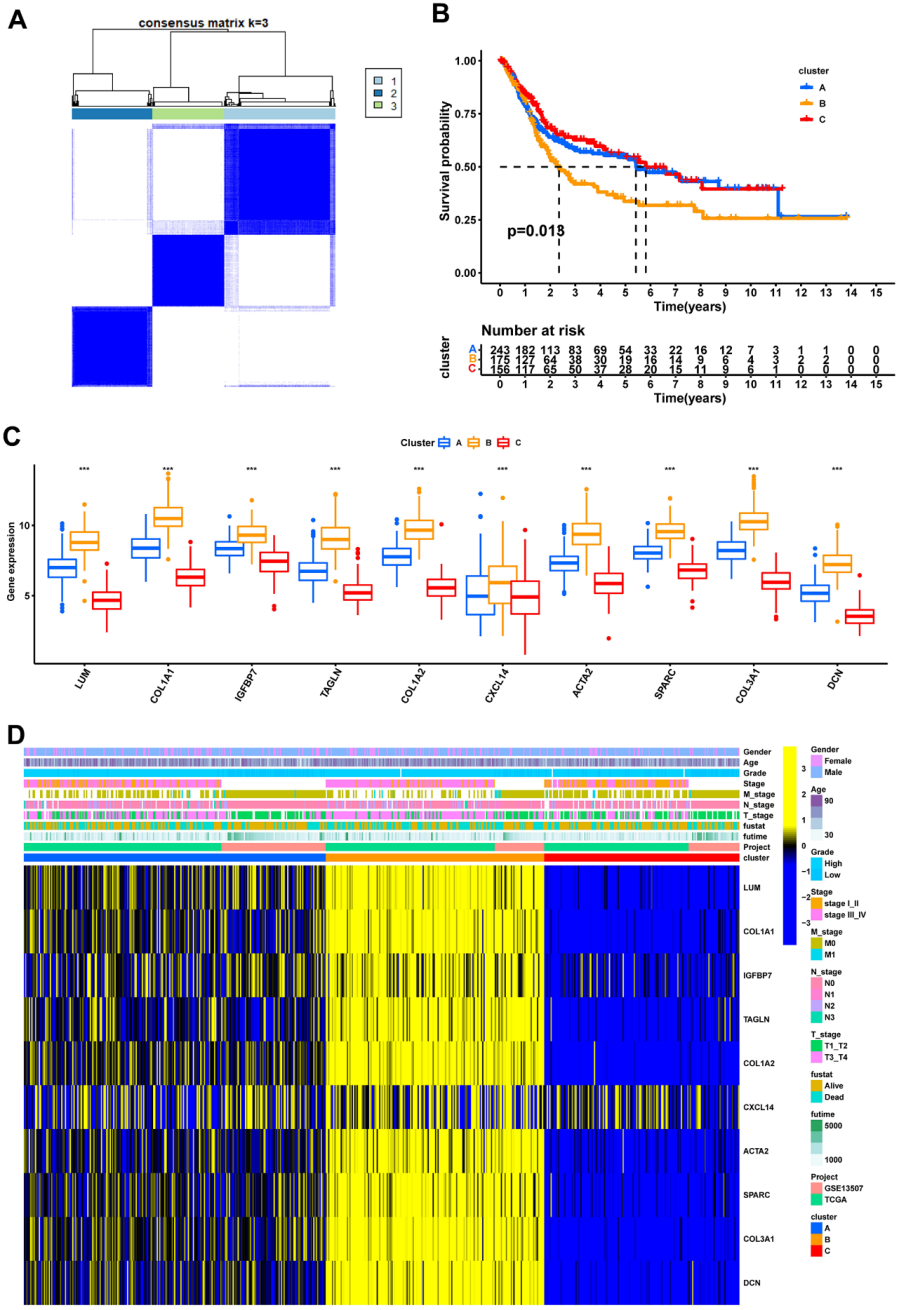
**Recognition of 3 fibroblast marker gene subtypes by consensus clustering analysis.** (**A**) Consensus matrix plots. K = 3 was determined as the optimal clustering number. (**B**) Kaplan-Meier survival analysis in clusters A, B, and C. (**C**) Differential expression of marker genes in fibroblast marker gene subtypes. (**D**) Heatmap of the marker gene expressions among clusters A, B, and C.

**Figure 5:**
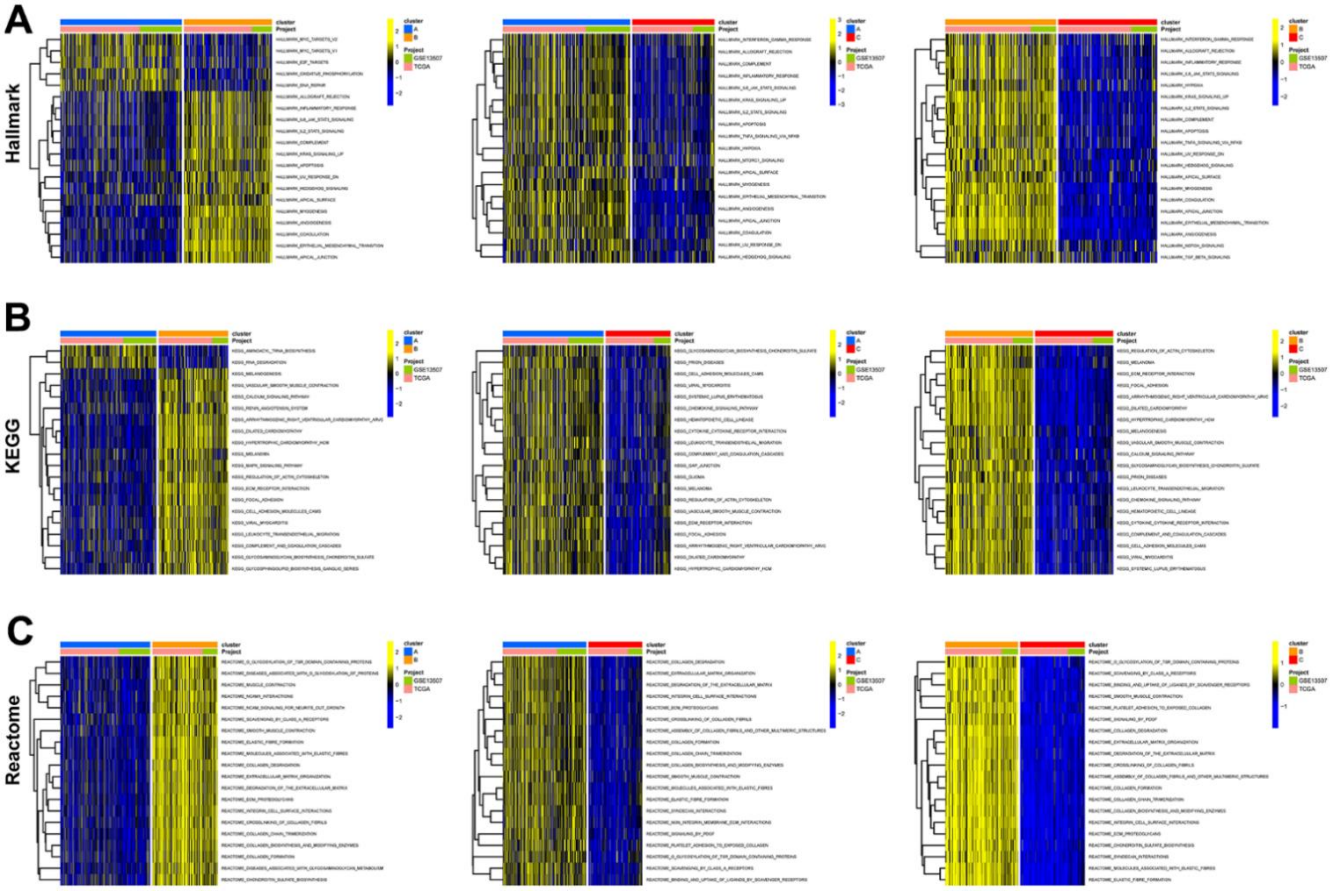
**Functional enrichment analysis of fibroblast marker gene subtypes.** (**A**) Enrichment analysis of 3 clusters in the HALLMARK pathway. (**B**) KEGG pathway. (**C**) Reactome pathway.

**Figure 6:**
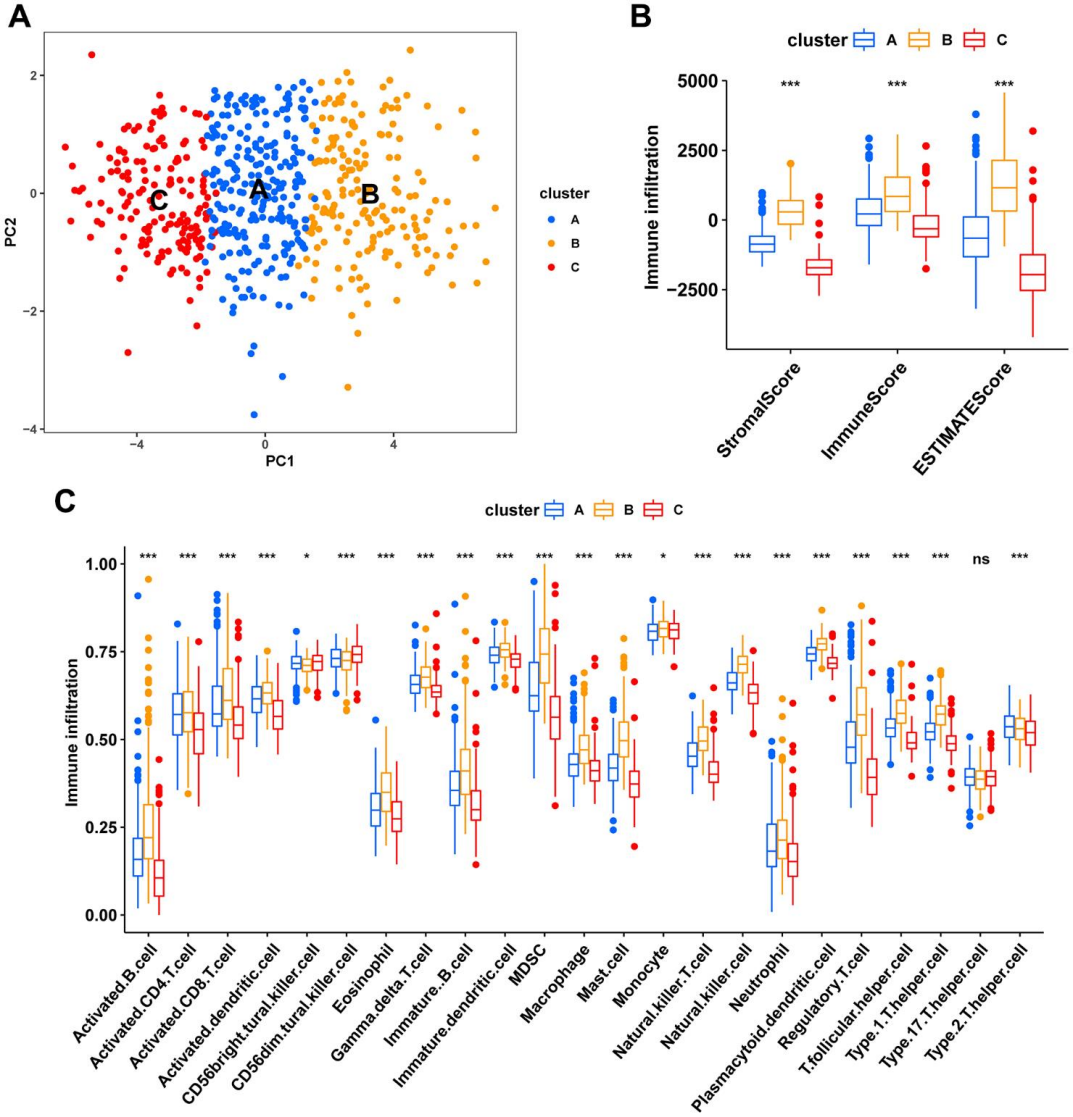
**Characteristics of the tumor immune microenvironment in fibroblast marker gene subtypes.** (**A**) PCA plots of the distribution of different subtype samples. (**B**) Differences in expression levels of stromal, immune, and ESTIMATE scores between clusters A, B, and C. (**C**) Differences in immune cell infiltration among different fibroblast marker gene subtypes.

Consensus clustering analysis revealed the presence of three subtypes based on the expression levels of marker genes (Figure 4A). Kaplan-Meier survival analysis demonstrated that the median OS of patients in cluster B was substantially lower than the other two subtypes (p=0.018) (Figure 4B). Figure 4C shows the comparison of gene expression levels among the three subtypes, where cluster B had the highest expression levels of all marker genes, followed by cluster A (p<0.001). The distribution of the remaining clinical features in the three subtypes is shown in Figure 4D. We further performed pathway enrichment analysis for each subtype and found that cluster B was mainly enriched in immune, metabolic, and tumorigenesis-related pathways (Figure 5A–5C). Afterwards, we investigated the correlation between each subtype and the immune microenvironment by using the ESTIMATE algorithm. The results revealed that the stromal score, immune score, and estimated score of cluster B exhibited significantly higher values than those of the other two groups. (p<0.001) (Figure 6A, 6B). The results of the ssGSEA algorithm were similar and indicated that the infiltration level of most immune cells was higher in cluster B, with Monocyte, Myeloid-derived suppressor cells (MDSC), and T cells being the major immune cell types (Figure 6C).

### Development of molecular subtypes and prognostic signature

**Figure 7:**
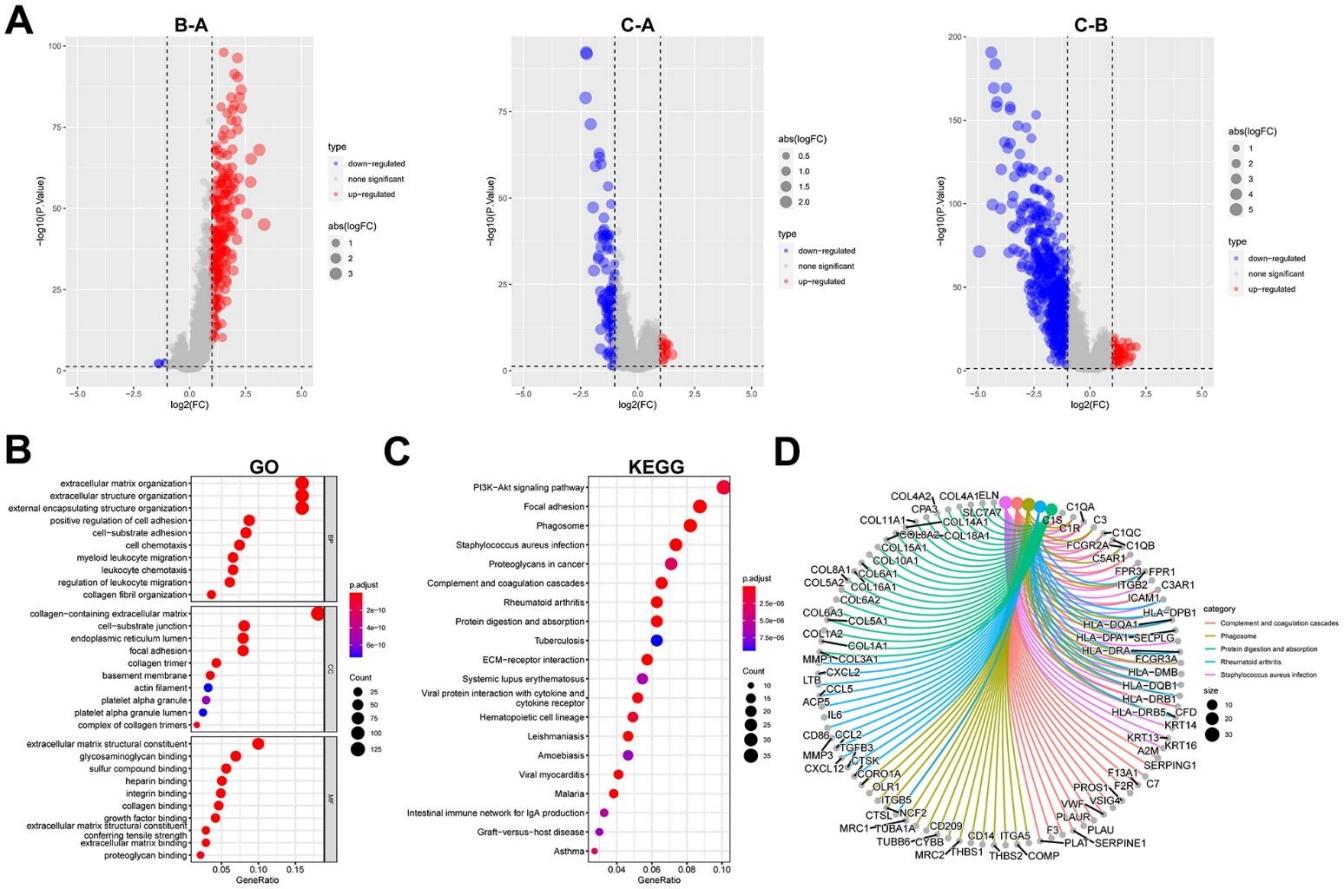
**Identification and functional enrichment analysis of DEGs between fibroblast marker gene subtypes.** (**A**) Volcano plots of differential expression of genes among clusters A, B, and C. The red dots represent upregulated genes and the blue dots represent downregulated genes. (**B**) Bubble plots of the GO terms of differential expression of genes. (**C**) Bubble plots of the KEGG pathways of differential expression of genes. (**D**) Correspondence between top 5 pathway and genes in KEGG.

**Figure 8:**
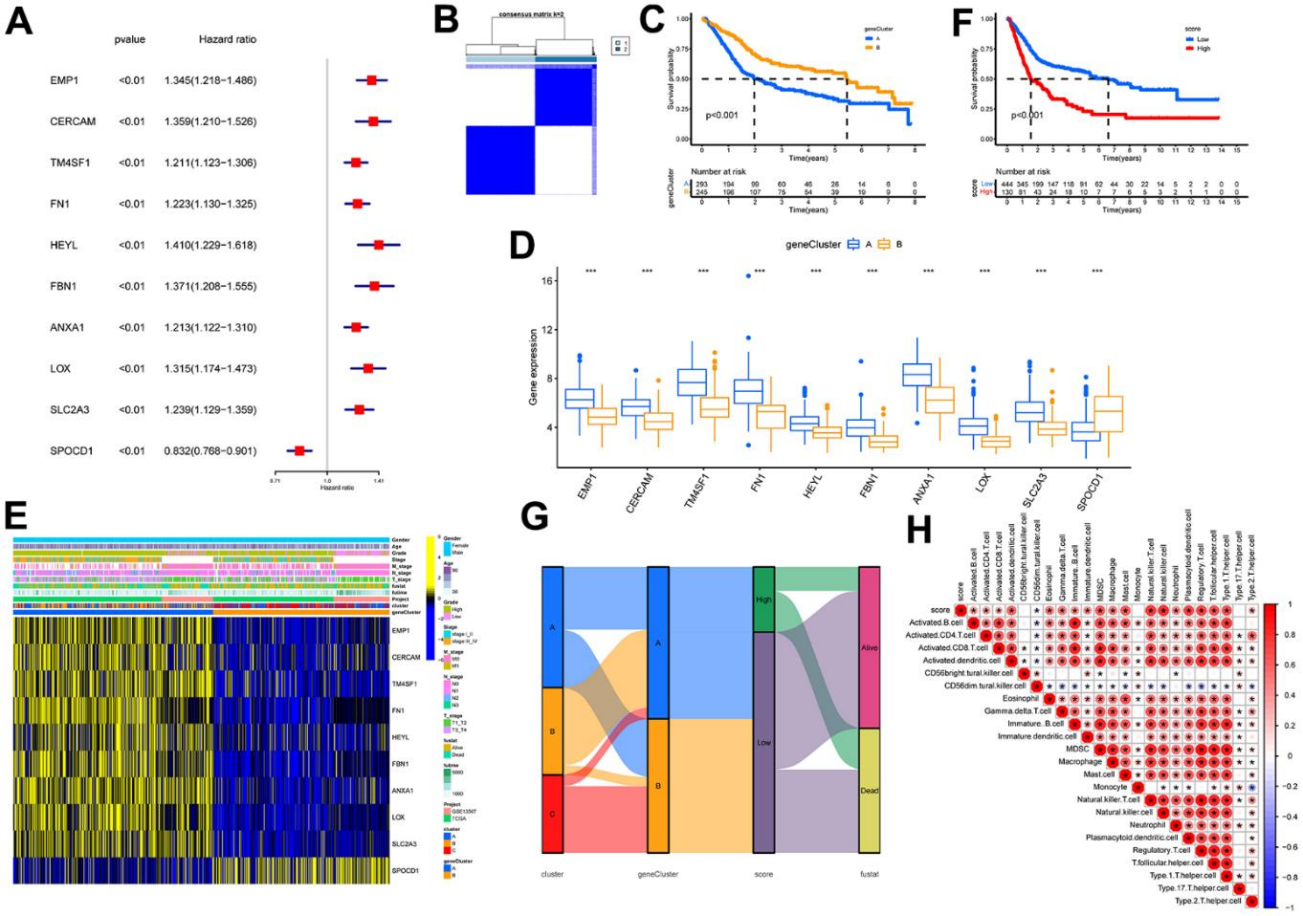
**Identification of molecular subtypes and prognostic signature.** (**A**) Forest plots for the univariate regression analysis of the 10 signature genes. (**B**) Consensus matrix plots based on signature genes. K = 2 was determined as the optimal clustering number. (**C**) Kaplan-Meier survival analysis in molecular subtypes A and B. Patients in subtypes A were related to a poorer prognosis than those in subtypes B. (**D**) Differential expression of signature genes in molecular subtypes. *** indicates p<0.001. (**E**) Heatmap of the signature gene expressions among molecular subtypes A and B. (**F**) Kaplan-Meier survival analysis between high- and low-score groups. Patients in high-score groups were related to a poorer prognosis compared to those in low-score groups. (**G**) Sankey diagram showing the dynamics of individual clusters concerning survival status. (**H**) Heat map of the correlation between prognostic signature score and immune cell infiltration. Red represents a positive correlation, blue represents a negative correlation; * indicates p<0.05.

A total of 723 DEGs were identified through a conjunction set following differential analysis among the subtypes (Figure 7A). Subsequently, GO/KEGG enrichment analysis revealed their association with an extracellular matrix structure, leukocyte migration, PI3K-Akt signaling pathway, and other functions (Figure 7B–7D). To detect prognosis-related genes, we performed univariate regression analysis and selected the top 10 genes with the smallest p-values as signature genes: EMP1, CERCAM, TM4SF1, FN1, HEYL, FBN1, ANXA1, LOX, SLC2A3, and SPOCD1. Among them, all but SPOCD1 were identified as prognostic risk factors (Figure 8A). Based on the signature genes, we classified the patients into two molecular subtypes (Figure 8B). Survival analysis revealed a significantly worse prognosis in patients belonging to gene cluster A (p<0.001) (Figure 8C), who also exhibited higher expression levels of signature genes (p<0.001). Furthermore, we compared the rest of the clinical characteristics (Figure 8D, 8E). We established a prognostic signature to comprehensively capture the differences between the two molecular subtypes. We calculated the signature scores through GSVA and divided the testing set samples into high- and low-score groups based on the optimal cutoff value. The median OS of patients in the high-score group was significantly lower than that of the low-score group (p<0.001) (Figure 8F). In repeated subgroup analysis, we observed that all patients in the high-score group belonged to the gene cluster A subtype, while most patients in the low-score group were in survival status. This indicates that the prognostic signature has good predictive value in BLCA (Figure 8G). Moreover, we found a significant positive correlation between risk score and multiple immune cell infiltrations (p<0.05), particularly MDSC, NKT cell, NK cell, and Treg cell (Figure 8H).

### Validation of the risk score in different clinical patho-characteristic subgroups

**Figure 9:**
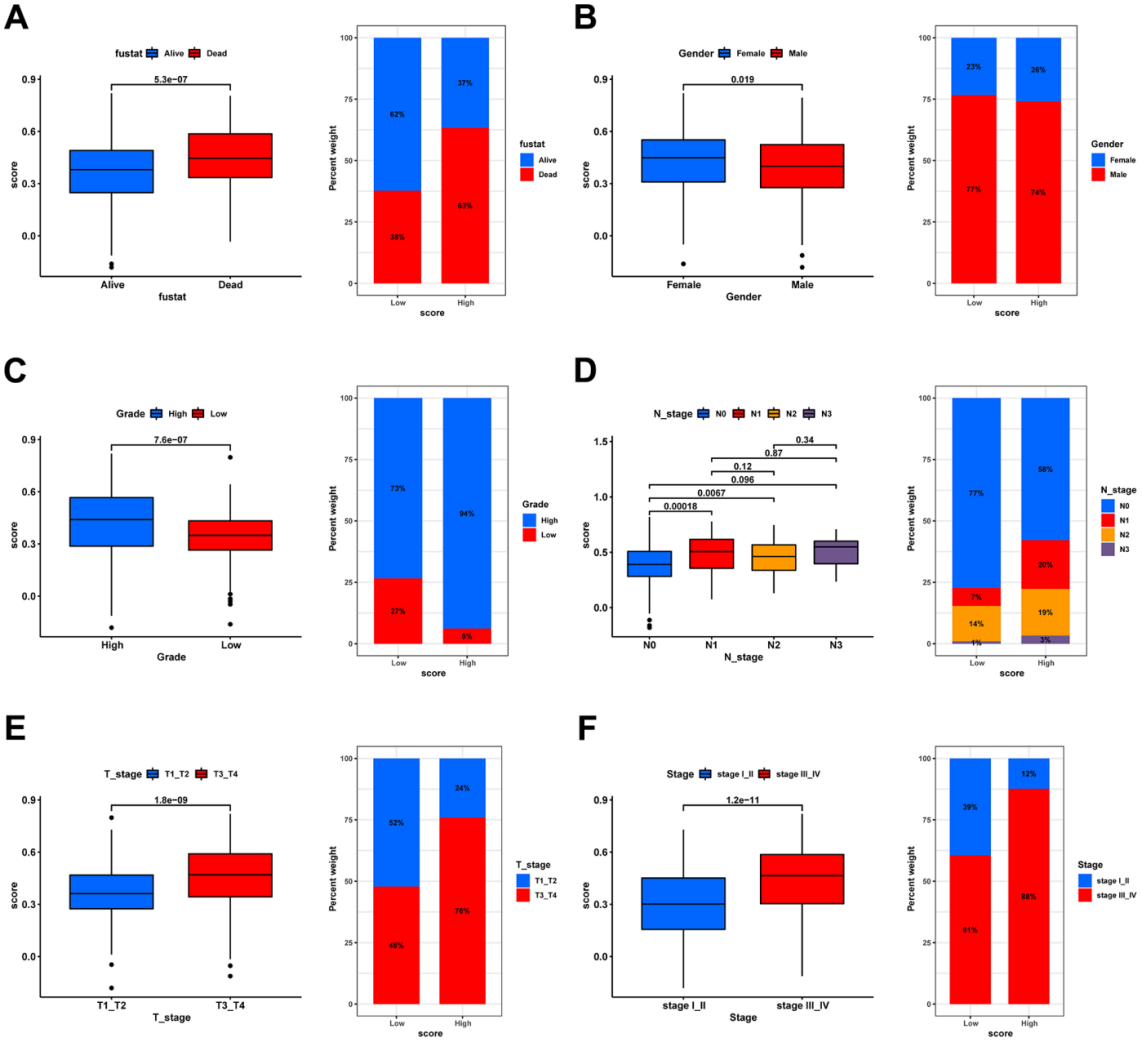
**Correlation analysis of risk scores with clinical characteristics.** Box plot for differences in risk score distribution (left) and bar plot for sample distribution in high- and low-risk groups (right) in different survival status (**A**), gender (**B**), grade (**C**), N stage (**D**), T stage (**E**), and tumor stage (**F**).

The clinical characteristics of the tumor are of significant importance in determining its prognosis. Therefore, we evaluated the predictive capacity of the prognostic signature in patients exhibiting disparate states, genders, grades, N stages, T stages, and stages, by contrasting the discrepancies in clinical characteristics between high- and low-risk groups. Significant differences in risk scores were observed among all subgroups based on clinical characteristics, as illustrated in Figures 9A–9F. (p < 0.05). Moreover, the proportions of patients with death, high-grade, lymph node metastasis, T3/4, and stage III/IV were substantially higher in the high-score group than in the low-score group. Hence, it is suggested that risk scores have excellent predictive potential for clinical outcomes.

### Estimation and validation of the relationship between risk scores and immunotherapy response

**Figure 10:**
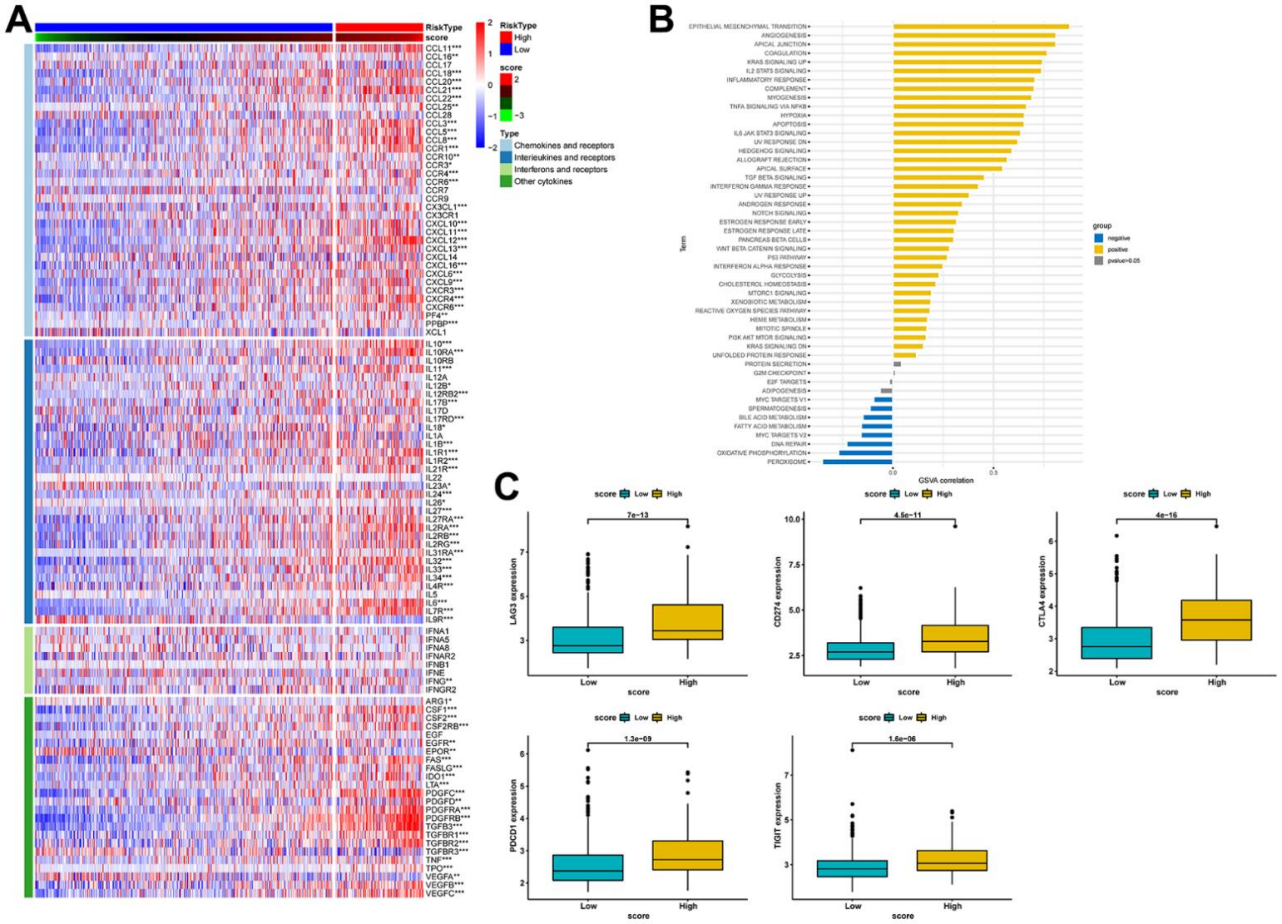
**Estimation of the relationship between risk scores and the tumor immune microenvironment.** (**A**) Heat map of cytokine expression in high- and low-score groups. * indicates p<0.05, ** indicates p<0.01, *** indicates p<0.001. (**B**) Correlations between signature score and HALLMARK pathway. (**C**) Expression analysis of LAG3, CD274, CTLA-4, PDCD1, and TIGIT between high- and low-risk groups.

Previous studies have reported that gene mutations can modify the response of cancer patients to immunotherapy and impact the selection of clinical drugs and treatment outcomes. Therefore, in this study, we compared gene mutation differences between the high- and low-score groups and found that missense mutation was the most common type of mutation in BLCA patients, and most genes were mutated more frequently in the high-score group (Supplementary Figure 2A–2C). We then analyzed the expression levels of cytokines and immune checkpoints in both groups. As shown in Figure 10A, most chemokines, interferons, and other cytokines, except interleukins, were notably over-expressed in the high-score group. The results of pathway enrichment analysis further confirmed that the risk score was positively correlated with tumorigenesis and multiple immune-related pathways, including EMT, angiogenesis, compliment, and IL-6/JAK/STAT3 signaling pathway (Figure 10B).

**Figure 11:**
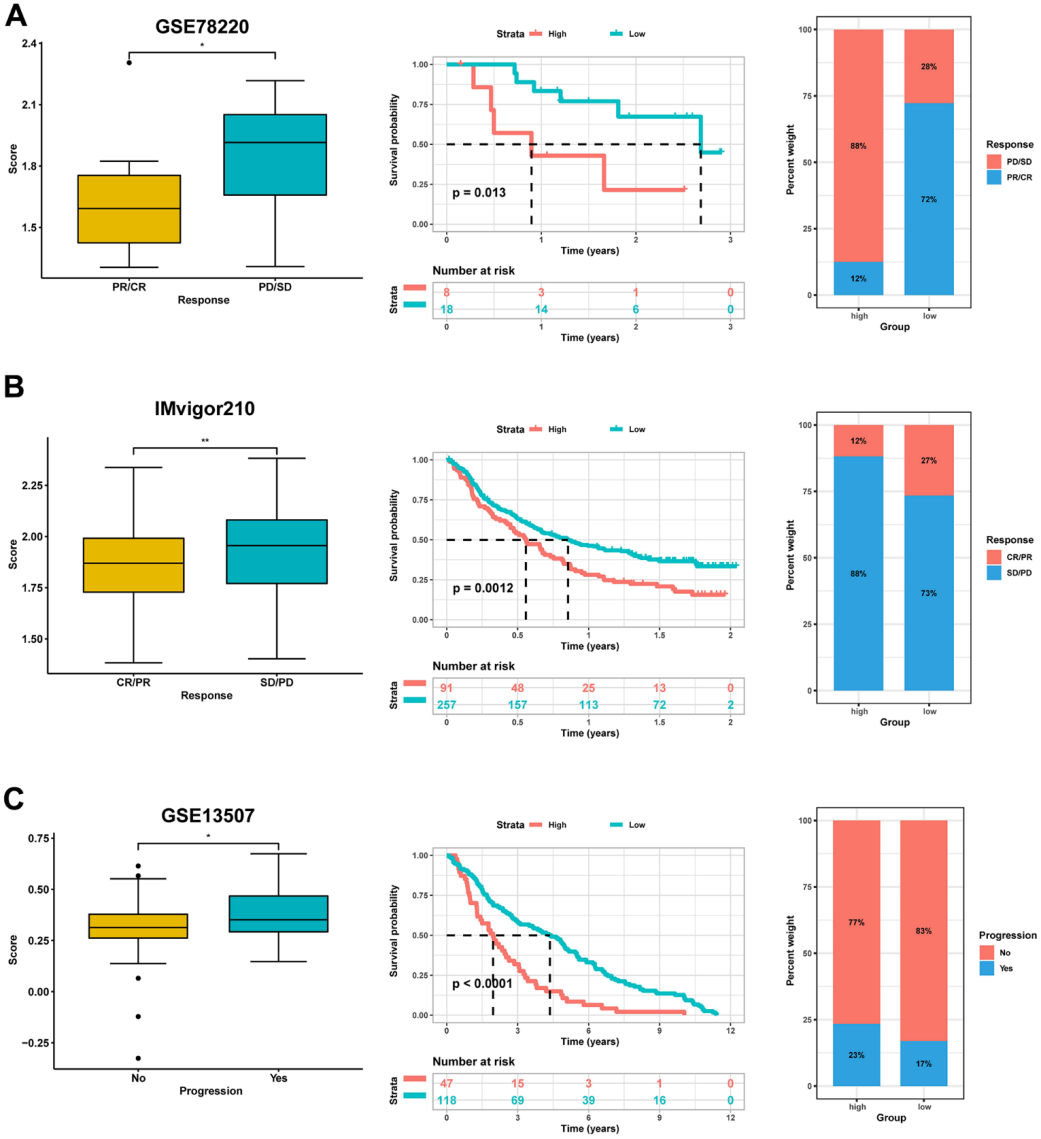
**Validation for the predictive role of risk score on the immunotherapeutic response.** (**A**) Kaplan-Meier survival analysis and distribution of immunotherapy responses of high- and low-score groups in GSE78220. (**B**) Kaplan-Meier survival analysis and distribution of immunotherapy responses of high- and low-score groups in IMvigor210 dataset. (**C**) Kaplan-Meier survival analysis and progress risk of high- and low-score groups in the GSE13507 dataset.

Similarly, our comparative analysis revealed that the expression levels of five immune checkpoints, LAG3, CD274, CTLA4, PDCD1, and TIGIT, were significantly elevated in the high-score group (p<0.001) (Figure 10C). Collectively, these results demonstrated that the risk score was associated with the immunotherapy response in BLCA patients. To further substantiate this finding, we investigated the predictive value of the risk score by assigning patients in the GSE13507, GSE78220, and IMvigor210 cohorts to low-risk and high-risk groups. The risk score was found to have predictive value for immune checkpoint inhibitors. It was observed that patients who responded to anti-PD1, anti-PDL1, and intravesical BCG immunotherapy had distinctly lower risk scores. In contrast, patients in the high-score group had significantly worse objective response rates to immunotherapy and were more inclined to poorer survival outcomes (p < 0.05). Additionally, we found that patients with high-score had a higher incidence of tumor progression compared to patients with low-score (Figure 11A–11C). Taken together, these findings strongly suggest that the risk score may serve as a promising marker for immunotherapy in BLCA patients, potentially determining a specific immune profile. Patients in the high-score group are more likely to tolerate immunotherapy.

### Prediction of potential sensitive drugs

**Figure 12:**
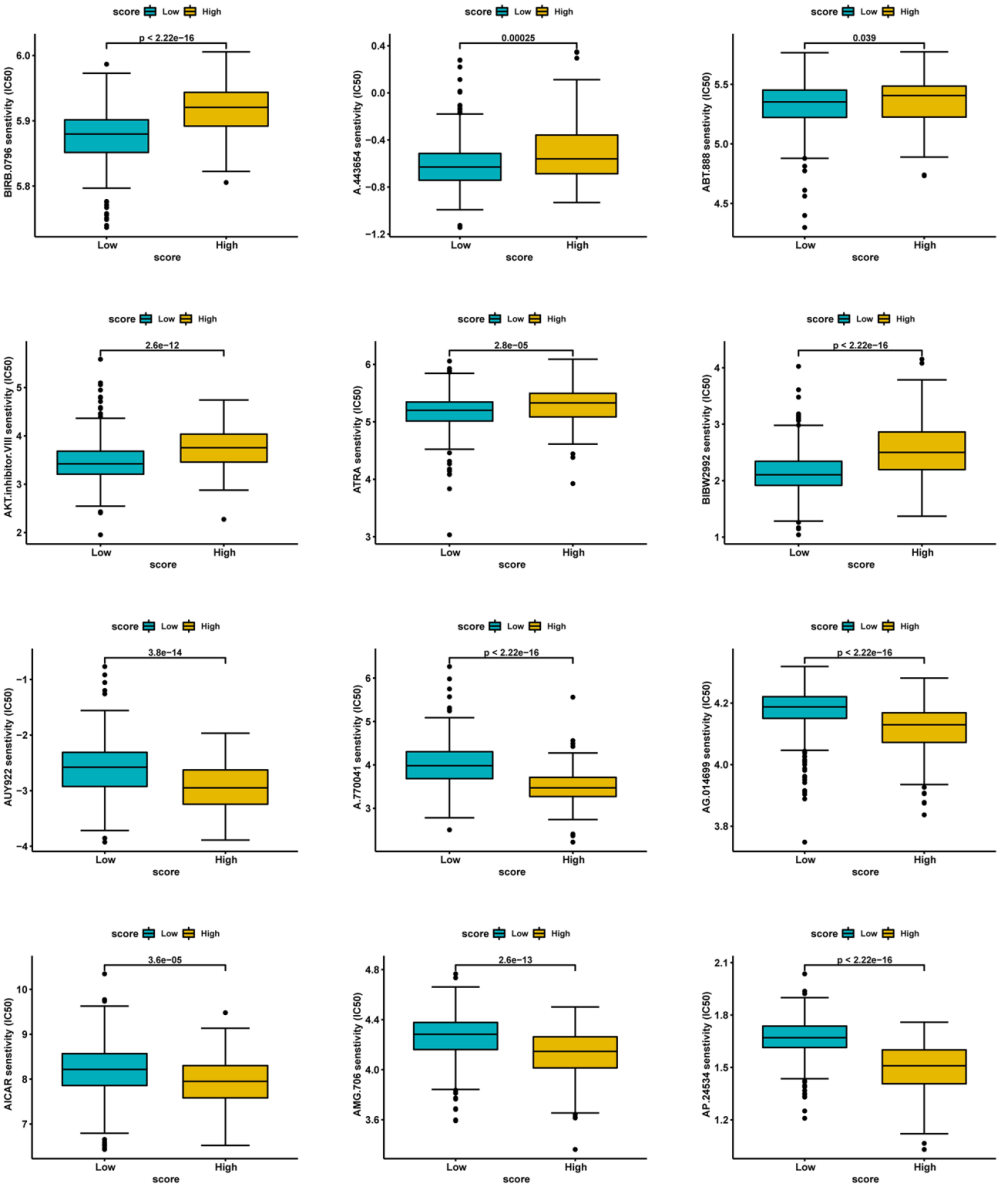
Prediction of potential sensitive drugs in high- and low-score groups.

Lastly, we investigated potentially sensitive chemotherapeutic agents for immunotherapy-tolerant patients. By comparing IC_50_ levels of chemotherapy drugs between the two groups, we found that patients in the low-score group exhibited lower IC_50_ values for anti-cancer drugs, including BIRB.0796, A.443654, ABT.888, AKT.inhibitor.VIII, ATRA, and BIBW2992. Patients with high scores were more likely to be sensitive to chemotherapy drugs, including AUY922, A.770041, AG.014699, AICAR, AMG.706, and AP.24534 (Figure 12).

### Validation of genes using IHC and *in vitro* assays

The RT-qPCR results revealed a statistically significant difference in gene expression between normal bladder uroepithelial cells and BLCA for the nine genes analyzed (Supplementary Figure 3). Furthermore, the expression patterns of most genes observed in the PCR experiments were consistent with those observed in TCGA transcriptome data. To validate these experimental results at the translational level, IHC experiments were performed (Supplementary Figure 4). The expression levels of the nine genes were found to be consistent between mRNA and protein levels.

## DISCUSSION

Despite extensive use in tumor patients, immunotherapy’s response rate is not as excellent as expected. One of the most influential factors in the occurrence of immunotherapy resistance is the tumor immune escape mechanism. Evidence suggests that antigen-presenting cells are crucial for T-cell activation and tumor immunity, and cancers can evade this immunity through immune editing mechanisms such as immune dominance, the absence of immune checkpoints, or downregulating antigen-presenting cells. Recent advancements in cancer research have highlighted the TME as a key determinant of patients’ responses to immunotherapy [30–32]. CAFs, essential components of the TME, regulate tumor proliferation, angiogenesis, invasion, metastasis, and treatment resistance in numerous malignancies. The application of scRNA-seq technology has revealed the molecular features and heterogeneity of various cell types within the TME, primarily focusing on immune cells [33, 34]. However, the investigation of stromal cells within the TME has been limited. Fibroblasts, which constitute the majority of stromal cells in the TME, play a vital role in anti-tumor immunity. At the initial stages of malignancy, fibroblasts can secrete TGFβ and hepatocyte growth factor, inducing the initiation of cancer within the normal human epithelium. Therefore, we employed a combination of bulk RNA-seq and scRNA-seq analyses to identify fibroblast-specific marker genes. Subsequently, we classified BLCA patients into three distinct molecular subtypes using these markers and developed and validated a prognostic signature to predict the survival outcome and responsiveness to immunotherapy in these patients.

Our findings demonstrate that the fibroblast-related prognostic signature is significantly associated with clinical characteristics and outcomes of BLCA patients. The high-score group was characterized by a higher grade, a more advanced stage, and an inferior OS. These results are consistent with previous studies that have linked fibroblast activity to poor cancer prognosis, but our study uniquely identifies specific marker genes and their implications in BLCA. Furthermore, the signature was validated across multiple datasets, demonstrating good stability in predicting OS. This prompted us to investigate the underlying mechanisms in greater depth. We determined ten genes (EMP1, CERCAM, TM4SF1, FN1, HEYL, FBN1, ANXA1, LOX, SLC2A3, and SPOCD1) that constituted the prognostic signature. HEYL, SLC2A3, and FBN1 were found to mediate tumor progression and chemoresistance mainly by facilitating angiogenesis and EMT [35–37], while CERCAM, ANXA1, and SPOCD1 promoted tumor progression through the PI3K/AKT and EGFR signaling pathways. The remaining genes were associated with immune cell infiltration and immunotherapeutic response [38–40]. After conducting GSVA analysis, we hypothesized that certain genes might contribute to the poorer prognosis observed in the high-score group. Additionally, studies have shown that OICR-9429 enhances the chemosensitivity of bladder cancer cells to cisplatin, increasing apoptosis rates and cisplatin cytotoxicity, thus holding significant clinical implications [41]. Remodelin, by inhibiting NAT10 expression, has been found to increase bladder cancer patients’ sensitivity to cisplatin, reducing the S phase population in the cell cycle, enhancing bladder cancer cell chemosensitivity to cisplatin, and inducing apoptosis [42]. This underscores the importance of discovering novel targets for chemoresistance in bladder cancer, representing a promising avenue of research.

Our comprehensive investigation into the relationship between the prognostic signature and the tumor immune microenvironment revealed a positive correlation between the risk score and the infiltration of multiple immune cells, including MDSC, NK cells, and Treg cells. While cytotoxic T cells are critical effector molecules in anti-tumor immunity, CAFs and Treg cells can create immunologic barriers that lead to cytotoxic T cell dysfunction or exhaustion during cancer progression [43, 44]. This suggests that patients in the high-score group may experience reduced anti-tumor activity. Further analysis showed that patients in the high-score group had significantly elevated levels of chemokine expression. The role of chemokines in the tumor immune response is bidirectional. Pro-tumorigenic immune cells can recruit immunosuppressive cells via chemokine-dependent infiltration at different tumorigenesis stages and immune cell activation states, thereby reinforcing pro-tumorigenic responses [45]. This phenomenon may partially contribute to the observed immunosuppression in the high-score group.

Immune checkpoint molecules are crucial for immune function and have significant clinical implications in immunotherapy. Motivated by discrepancies in the tumor immune microenvironment, we explored the potential of the prognostic signature to predict responses to immunotherapy. We observed that patients in the high-score group had elevated levels of immune checkpoints but exhibited poorer response rates to immunotherapy. These findings suggest immune exhaustion as a possible mechanism behind the resistance to immunotherapy in the high-score group. This is particularly innovative, as it indicates specific molecular targets for overcoming immune resistance in BLCA patients.

The purpose of the signature is to enhance patients’ responses to immunotherapy and to identify methods of overcoming immune resistance. We predicted potential target drugs and found that both high- and low-scoring groups might benefit from six different anti-tumor drugs, including VEGFR inhibitors, AMPK activators, and PARP inhibitors, among others. For instance, Li et al. found that VEGFR and EGFR inhibition increases epithelial characteristics and chemotherapy sensitivity in mesenchymal bladder cancer cells [46]. Zhou et al. revealed that artesunate induces autophagy-dependent apoptosis through upregulating ROS and activating AMPK-mTOR-ULK1 axis in human bladder cancer cells [47]. Moreover, Bhattacharjee et al. discovered that PARP inhibitors enhance and synergize with cisplatin to inhibit bladder cancer cell survival and tumor growth [48]. Collectively, these findings could serve as a clinical reference for selecting drugs, although these drugs need further confirmation through clinical studies before they can be applied.

The present study has some limitations that can be addressed in future work. Firstly, we constructed the prognostic signature using the ten most relevant prognostic genes, but other prognostic-related genes were not included, which may introduce some bias. To avoid selection bias and enhance the accuracy of the analysis, future validation of this signature will require more prospective and multicenter BLCA cohorts. Secondly, the sample size of scRNA-seq data is relatively small, and clinicopathological characteristic data were unavailable. Bulk RNA-seq analysis of a larger sample size partially compensates for this limitation and ensures the accuracy of the prognostic signature. Thirdly, it should be noted that the mechanisms discussed in this study and the sensitive drug predictions are primarily based on the analysis of public databases and theoretical descriptions. Further *in vivo* and *in vitro* experiments are required to confirm the predictive value and potential mechanisms of this signature. Lastly, biomarker-driven prospective clinical trials are needed to determine treatment decisions for advanced-stage tumors. Therefore, our next objective is to conduct a comprehensive study aimed at elucidating the potential mechanisms underlying this signature, with the ultimate goal of its clinical application.

## CONCLUSIONS

After analyzing scRNA-seq and bulk RNA-seq data, we have successfully developed and validated a risk signature associated with fibroblasts. This signature can serve as an independent prognostic indicator for patients diagnosed with BLCA. The signature is reliable and robust and can accurately predict the outcomes for BLCA patients. This can help clinicians make more informed and rational decisions related to treatment.

## Supplementary Material

Supplementary Figures

Supplementary Table 1

Supplementary Table 2
