# Supplementary material for: Integrating single-cell RNA-seq to identify fibroblast-based molecular subtypes for predicting prognosis and therapeutic response in bladder cancer
Source: Aging (Albany NY). 2024 Jul 18;16(14):11385–408. doi: 10.18632/aging.206021 (PMC11315389; doi:10.18632/aging.206021)
Supplement: Supplementary Figures [file aging-16-206021-s001.pdf]

SUPPLEMENTARY FIGURES

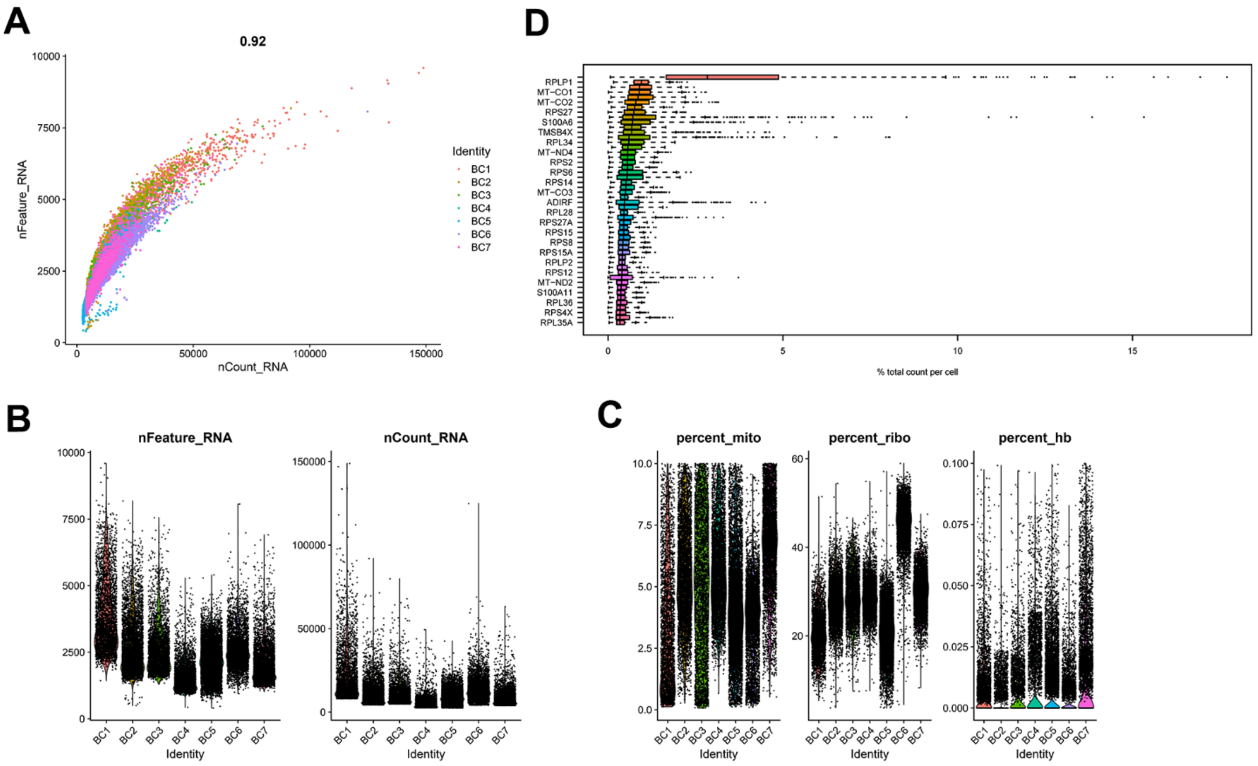

Supplementary Figure 1. Quality control of scRNA-seq data. (**A**) Correlation between nFeature_RNA and nCount_RNA. (**B**, **C**) Information of filtered scRNA-seq data from 7 BLCA samples. (**D**) The top 25 genes with the highest percentage of cellular expression.

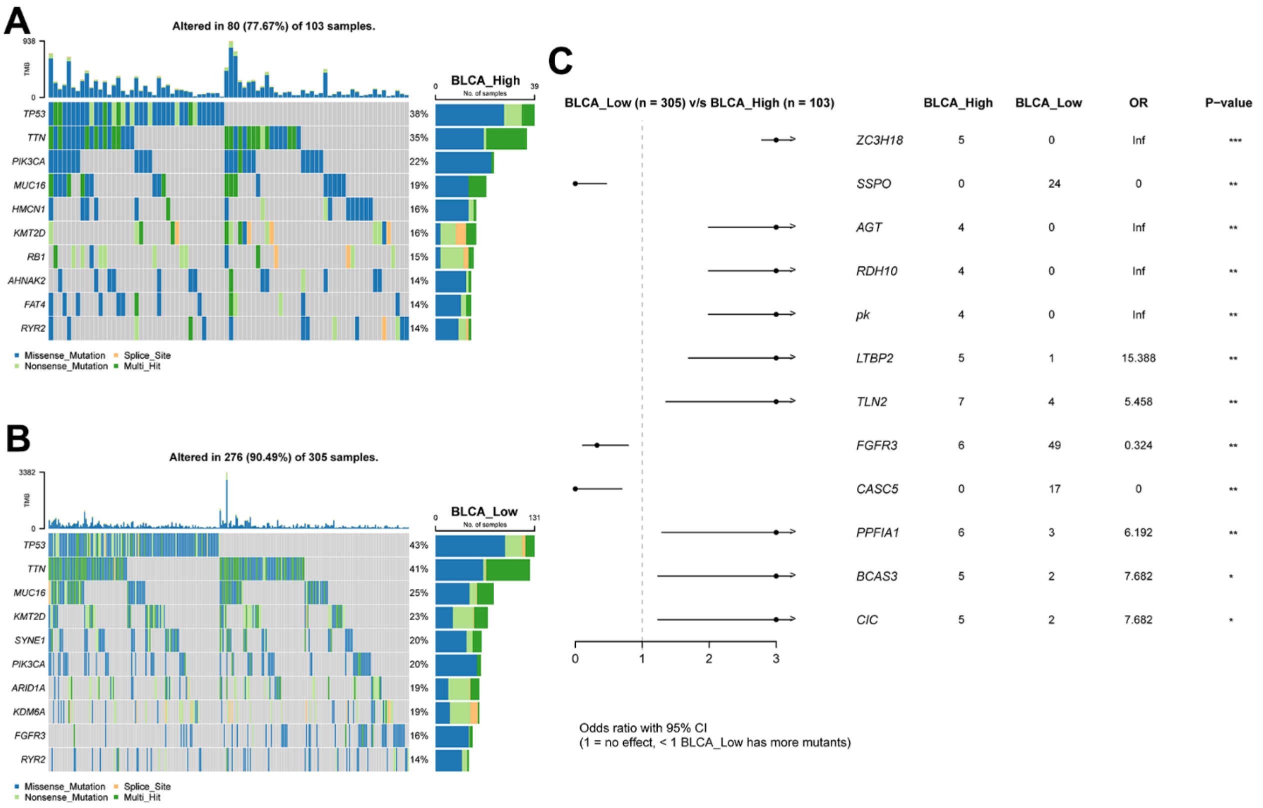

Supplementary Figure 2. Gene mutations in the high- and low-score groups. (**A**, **B**) Distribution of gene mutations in the high- and low-score groups. (**C**) Comparison of the gene mutations in the high- and low-score groups.

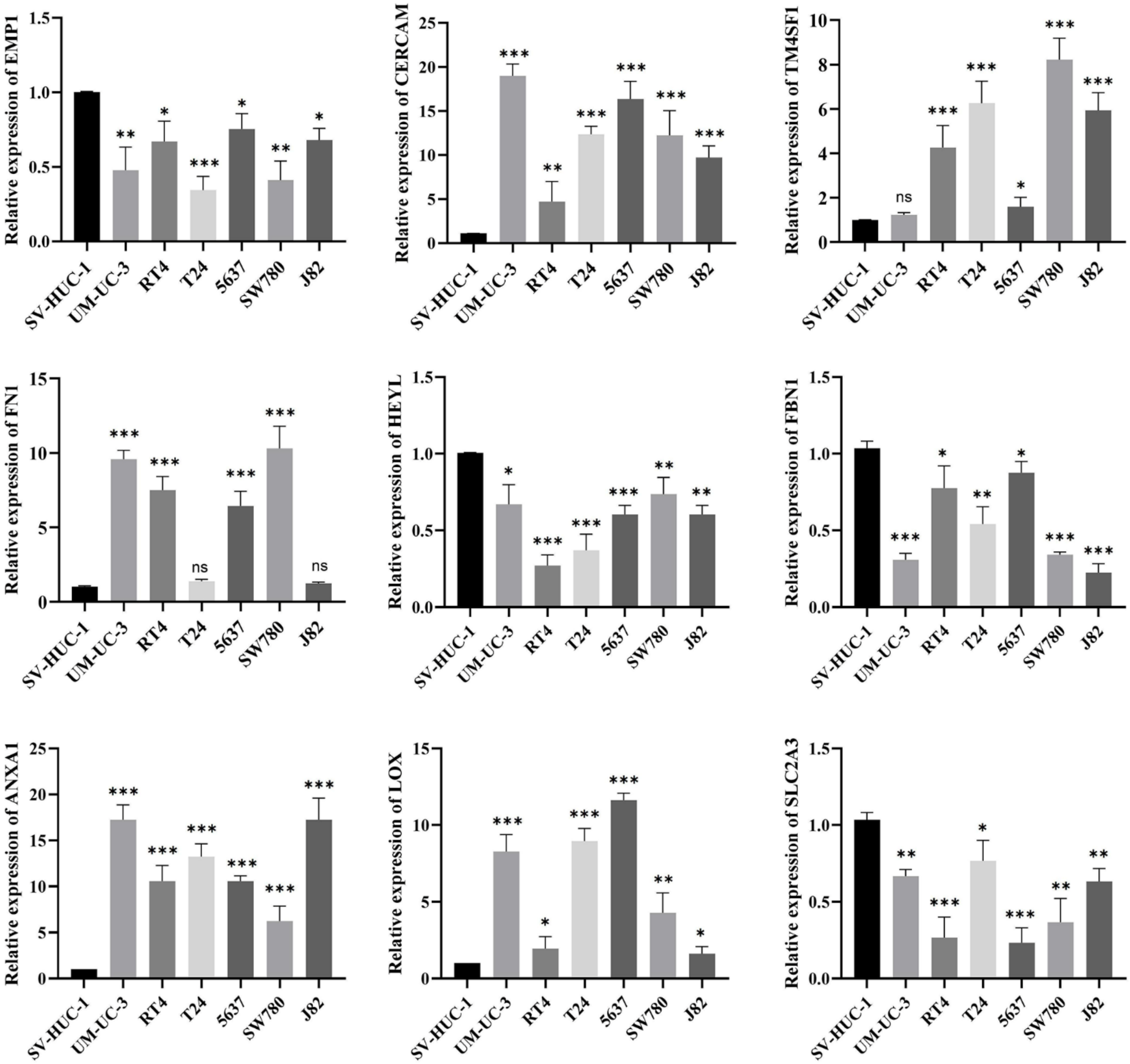

Supplementary Figure 3. mRNA expression levels of model genes in bladder uroepithelial cell line (SV-HUC-1) and BLCA cells (UM-UC-3, RT4, T24, 5627, SW780, and J82).

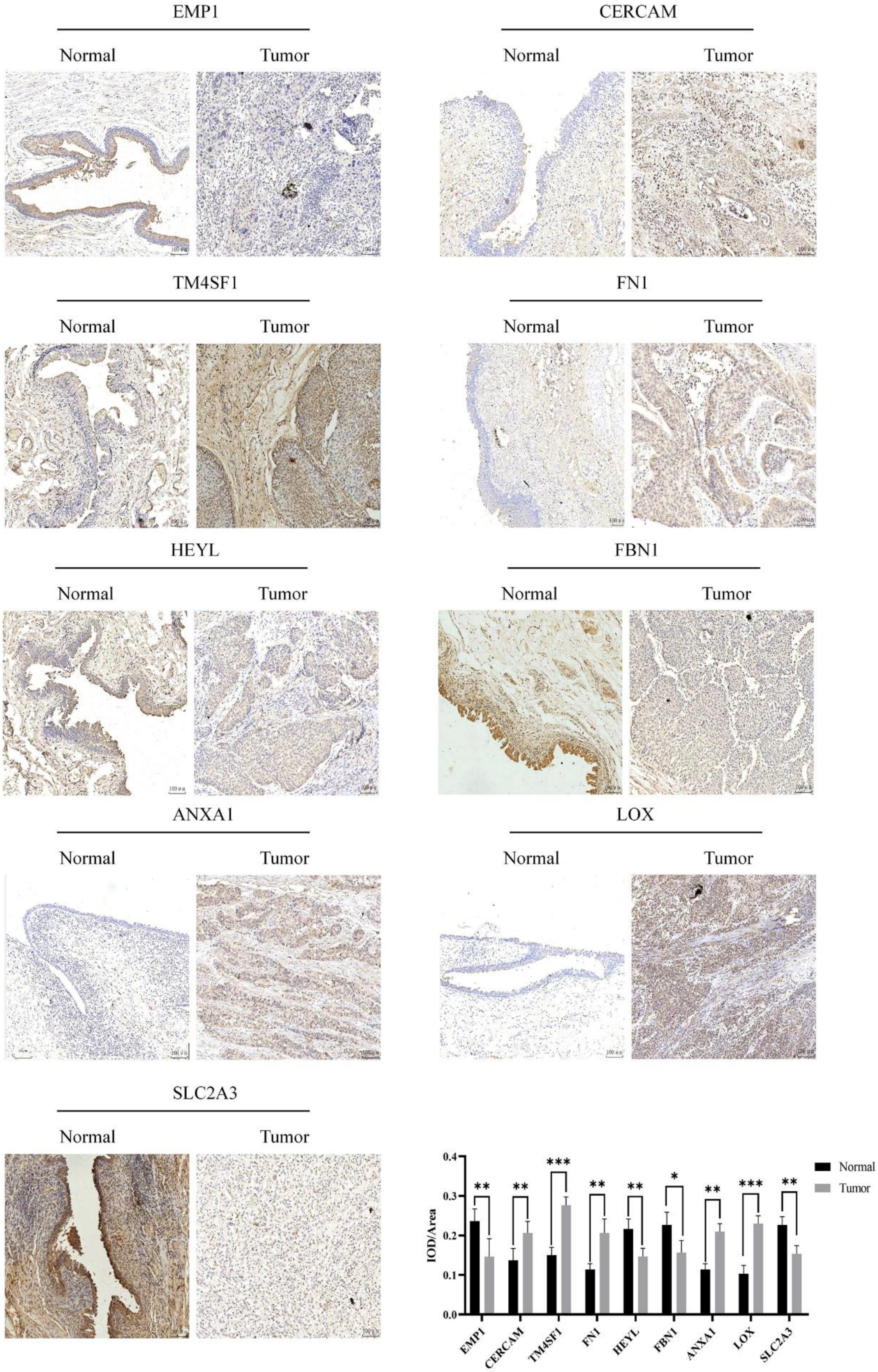

Supplementary Figure 4. Reprehensive image of the expression of model genes in tumor and paired normal samples revealed by IHC.
