# Supplementary material for: Integrating single-cell RNA-seq to identify fibroblast-based molecular subtypes for predicting prognosis and therapeutic response in bladder cancer
Source: Aging (Albany NY). 2024 Jul 18;16(14):11385–408. doi: 10.18632/aging.206021 (PMC11315389; doi:10.18632/aging.206021)
Supplement: Supplementary Table 1 [file aging-16-206021-s002.pdf]

SUPPLEMENTARY TABLES

Supplementary Table 1. Primer sequences in RT-qPCR.

	Forward primer	Reverse primer
EMP1	GTGCTGGCTGTGCATTCTTG	CCGTGGTGATACTGCGTTCC
CERCAM	GAGCCCAGGTTCTACCCAGAT	GCAGAGTCTGATTGTTGGTCA
TM4SF1	TGCATCGGACATTCTCTGGTG	GTTCCAGCCCAATGAAGACAA
FN1	CGGTGGCTGTCAGTCAAAG	AAACCTCGGCTTCCTCCATAA
HEYL	GGAAGAAACGCAGAGGGATCA	CAAGCGTCGCAATTCAGAAAG
FBN1	TTTAGCGTCCTACACGAGCC	CCATCCAGGGCAACAGTAAGC
ANXA1	GCGGTGAGCCCCTATCCTA	TGATGGTTGCTTCATCCACAC
LOX	CGGCGGAGGAAAACTGTCT	TCGGCTGGGTAAGAAATCTGA
SLC2A3	GCTGGGCATCGTTGTTGGA	GCACTTTGTAGGATAGCAGGAAG
GAPDH	CCCACTCCTCCACCTTTGAC	CCACCACCCTGTTGCTGTAG
